# Molecular docking, dynamics and *in vitro* analysis of multi-target inhibitors for *Clostridioides difficile*

**DOI:** 10.6026/973206300200039

**Published:** 2024-01-31

**Authors:** Nikita Chordia Golchha, Hasanain Abdulhameed Odhar, Anand Nighojkar, Sadhana Nighojkar

**Affiliations:** 1 School of Biotechnology, Devi Ahilya University, Takshashila Campus, Khandwa Road, INDORE-452001, India; 2 Department of Pharmacy, Al-Zahrawi University College, Karbala, Iraq; 3 Maharaja Ranjit Singh College of Professional Sciences, Hemkunt Campus, Khandwa Road, Indore, 452001, India; 4 Mata Gujri College of Professional Studies, A.B. Road, Indore, 452001, India

**Keywords:** *Clostridioides difficile*, inhibition, pathogen, compounds, *in silico*, molecular docking, dynamics, *in vitro*

## Abstract

The opportunistic pathogen, *Clostridioides difficile* owes its extreme pathogenicity for its ability to develop antibiotic resistance and recurrent infections. The current antibiotics used for the treatment are showing declining sensitivity and rising antibiotic resistance. Therefore, it is of interest to develop the anti-clostridial drugs to overcome these issues. Hence, we have explored ZINC library to find the suitable lead compounds against five target proteins of *C. difficile*. Multistep virtual screening is performed to find the suitable compounds that are checked for their stability using molecular dynamics and are validated *in vitro* against *C. difficile*. In our study, five compounds viz., ZINC64969876, ZINC13641164, ZINC13691348, ZINC5554596 and ZINC3894278 that inhibit HisC, Spo0A, PdcA, DAHP synthase and cyclic-di GMP proteins, respectively have been identified. Further, these compounds were tested *in vitro* against four different isolates of *C. difficile* and all of them were found to inhibit the pathogen. However, to use these compounds as anti-clostridial drugs, further testing needs to be done. The selected compounds from our study are reported for the first time as antimicrobial agents against *C. difficile*.

## Background:

*Clostridioides difficile* (*C. difficile*), causes wide range of diseases from diarrhoea to pseudomembranous colitis (PMC) [1-2]. It is the most prevalent pathogen causing hospital associated infections and is declared as an urgent threat by CDC (Center for Disease Control and Prevention) [3-4]. The main risk factors associated with this infection are antibiotic usage; but hospitalization, higher age, immunosuppression and severe co-morbidities can be other factors [5-6]. In addition, other reported risk factors include inflammatory bowel disease, enteral feeding, gastric acid suppression and cirrhosis [7- 8]. Toxins and endospores are the main virulence factors linked to the spread of disease [9]. *C. difficile* spores survive in the dormant states till favourable condition, and then they germinate in the gut to produce vegetative cells that produce toxin. Endospores are the major cause of spread and recurrence of *C.difficile* infection [10].

In the era of on-going pandemic of COVID 19, the usage of antibiotics has been tremendous, that leads to exponential increase in *C. difficile* infection (CDI) rates [11-12]. The antimicrobials used for treating CDI include metronidazole, fidaxomicin and vancomycin [13- 14]. Of these, metronidazole is a non-FDA-approved medication and is no longer advised for CDI. Fidaxomicin does not perform better in individuals with infection of hyper virulent strains. Therefore, only vancomycin is the preferred FDA approved drug for the treatment [15-16]. As the therapeutic antimicrobial options are limited for CDI due to its rising antibiotic resistance and declining sensitivity, there is an urgent need for the development of novel anti-clostridial drugs [17,18, 19,20,21]. Five proteins of C difficile that were reported as drug targets were used [22]. Therefore, it is of interest to report the molecular docking, dynamics and *in vitro* analysis of multi-target inhibitors for *Clostridioides difficile*.

## Methodology:

## Homology modelling:

The structures of all five drug targets namely HisC, Spo0A, PdcA, DAHP synthase and cyclic-di GMP are modelled using MODELLER 9v8 [23]. MODELLER was run using protocol given by Reddy *et al.*, 2015 [24]. The modelled protein structures were selected using lowest DOPE scores and are verified using online SAVES (Structural Analysis and Verification Server) server [25, 26,27].

## Ligand preparation and analysis of drug likeliness:

The 3D structure of drug like was downloaded in 3D format from ZINC database [28]. Approximately 11 million compounds were downloaded from ZINC database. These compounds were virtually evaluated using open source software DruLiTo, for their drug like property [29]. DruLiTo filters, namely Lipinski's rule, Veber rule, Quantitative Estimate of Drug-likeliness (QED), Ghose filter, BBB rule, CMC 50 like rule and MDDR like rule were used to study ADME profile of the compounds. The threshold values of the filters were kept at default and 18000 compounds which follow all the rules of drug likeliness were further screened for their toxicity.

## Toxicity prediction:

**Figure 1:**
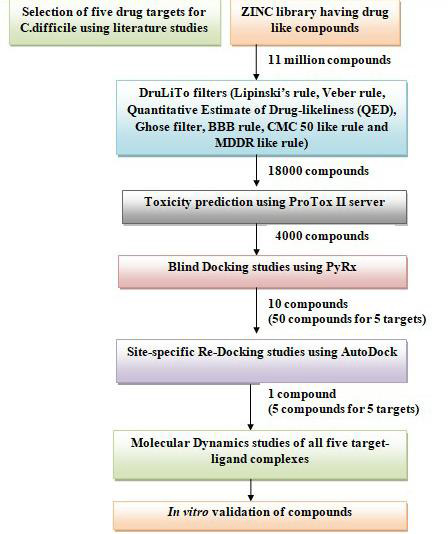
Flowchart depicting the methodology used.

*in silico* toxicity evaluation is a crucial step for better lead compound selection. It can be done computationally because of accuracy, rapidity and can provide results of any compound. For toxicity prediction we have used ProTox-II server which results in 4000 compounds [30]. Here, we have considered only class V and VI compounds for further screening. Figure 1 shows the flowchart depicting the methodology used in the current study.

## Molecular docking:

It is the crucial step of drug discovery process that is used to analyze the conformation and orientation of compound into the binding site of a target. The modeled protein structures of all five targets were blindly docked with the filtered 4000 ZINC compounds using AutoDock Vina of PyRx 0.8 using default settings [31]. Preparation of input files in pdbqt file format, energy minimization and virtual screening using vina wizard is done according to the protocol given by Dallakyan and Olson, 2015 [32]. The best 10 hits with lowest binding energy and more number of hydrogen bonds for each target were obtained and subjected to site specific docking using AutoDock 4.2 [33]. Active site for each target is identified using active site predictor [34]. Analysis and visualization of the docked molecule was done using PyMol software [35].

## Molecular dynamics (MD):

To confirm and calculate the stability, fold and interactions of the best docked complex, a 50 ns molecular dynamics simulation of the entire five target-ligand complex was carried out using the GROMACS v5.0.4 software package [36]. The topology file of protein and ligand was generated using all atoms CHARMM36 force field and Swiss-Param, respectively [37-38]. The complex was then placed in a cubic box with a minimum distance of 10 Å from the center to the box edge and the solubilization was performed using the TIP3P (transferable intermolecular potential with 3 points) water model. The system was neutralized by adding required number of Na+/Cl- ions and energy was minimized using steepest descent algorithm. Equilibration simulations were performed under constant NVT and NPT ensembles for 100 ps each, temperature was set at 300K and pressure at 1 bar. Finally, the production run for all the complexes was started at 50 ns. The results were analyzed by calculating root mean square deviation (RMSD), root mean square fluctuation (RMSF), hydrogen bonds, total energy, radius of gyration (Rg) and solvent accessible surface area (SASA).

## Antimicrobial assay:

(A) To test the selected compounds against *C. difficile*, four isolates of *C. difficile* have been used. Agar well diffusion assay and Minimum Inhibitory Concentrations (MICs) was done according to Clinical Laboratory and Standards Institute (CLSI) criteria for anaerobes [39]. For agar well diffusion assay, antimicrobial susceptibility testing was done on Brucella agar plates with a bacterial inoculum in Brain heart Infusion (BHI). Six wells (6 mm) were cut into each inoculated agar plate and a 100 µl aliquot of each diluted solution (50 µg/ml, 100 µg/ml, 150 µg/ml, 200 µg/ml, 250 µg/ml and control (DW) was pipetted into each well. The plates were then incubated in an anaerobic chamber at 37°C for 48 h. After incubation, zones of growth inhibition were measured to the nearest millimetre.

(B) Broth dilution method was used to find the minimum concentration of compound that will inhibit the growth of a microorganism. The strains were grown in BHI broth and incubated anaerobically at 37°C for 48 h. Compounds to be tested were diluted with distilled water to get the different concentrations of 100µg/ml, 50µg/ml, 25µg/ml, 12.5µg/ml and 6.25µg/ml of test compound. Tubes containing 1ml of BHI broth was added with 1ml of each concentration of tested compounds. The tubes were then inoculated with 20µl of bacterial culture. Control tubes were prepared using 1ml of broth with 20 µl of inoculums, and their optical density is compared with the test compounds. After incubation, optical density (OD) at 600 nm was measured and percentage inhibition was calculated using the formula:

% Inhibition =(OD(Control)- OD(Sample))/(OD(Control)) x 100

## Results and Discussion:

**Figure 2:**
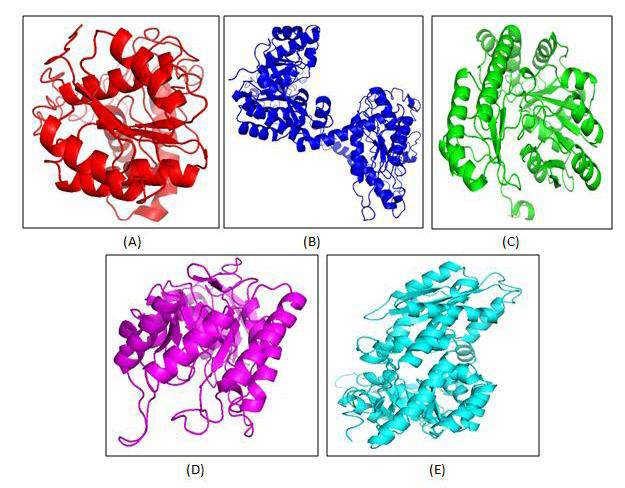
Cartoon representation of all five modeled protein structure (A) sporulation transcription factor (Spo0A), (B) c-di-GMP phosphodiesterase (PdcA), (C) histidinol-phosphate transaminase (HisC), (D) 3-deoxy-7-phosphoheptulonate synthase (DAHP synthase) and ( E) bifunctional diguanylate cyclase/ phosphodiesterase (cyclic-diGMP).

Protein modeling of all five drug targets namely HisC, Spo0A, PdcA, DAHP synthase and cyclic-di-GMP performed using MODELLER, yielded a full length model of these targets. Figure 2 shows the cartoon representation of all the five modeled proteins. The structures are validated using online SAVES server whose data validate our modeled structure. All the modeled protein targets show good ERRAT quality factor for protein conformation. In addition to this, more than 94 percent of residues fall in core, allowed and generously allowed region of Ramachandran plot, thereby validating our structures.

The library of compounds downloaded from ZINC database contains approximately 11 million compounds, which are filtered based on several parameters as shown in Table 1. The initial step of virtual screening is drug likeliness. Generally, only Lipinski's rule is considered for drug likeliness but several reports indicated that many promising drugs do not follow Lipinski's rule and alone it is not sufficient to prevent the potential exclusion of effective substances [40- 41]. Here we have considered seven filters that can make them quite efficient and effective and are more likely to be transformed into drugs. This resulted into approximately 18000 compounds which were further filtered based on their toxicity.

**Figure 3:**
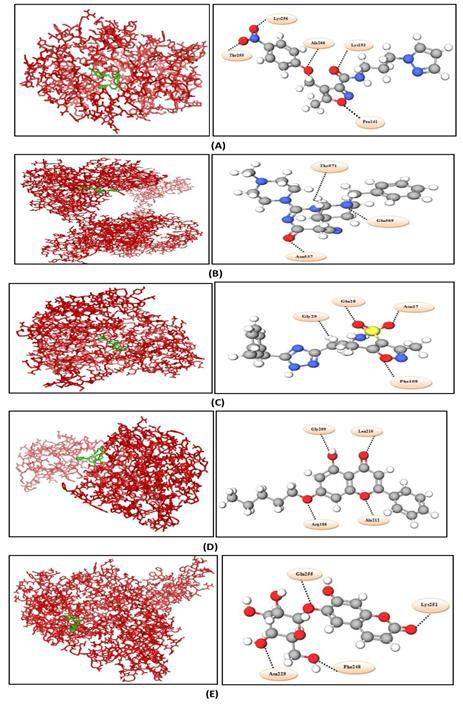
Binding orientation of all five docked complexes. Macromolecule is shown in red color and ligand in green color. (A) Sporulation transcription factor (Spo0A) and ZINC13641164, (B) c-di-GMP phosphodiesterase (PdcA) and ZINC13691348, (C) histidinol-phosphate transaminase (HisC) and ZINC64969876, (D) 3-deoxy-7-phosphoheptulonate synthase (DAHP synthase) and ZINC5554596, (E) bifunctional diguanylate cyclase/ phosphodiesterase (cyclic-diGMP) and ZINC3894278.

Toxicity predictions were done using Protox2, in which we have considered only Class V and Class VI compounds. Class V and VI compounds have very less chances of toxicity which can be further useful in clinical trial studies. After applying all these filters, we get approximately 4000 compounds which were docked with each target molecule. Initial virtual screening using PyRx software generated nine different conformations for each ligand which are classified by binding affinity (kcal/mol). The top 10 ranked compounds for each target molecule were selected. Active sites of all the targets were predicted using active site predictor. Then they were site specifically docked with top 10 ranked compounds using AutoDock 4.2. In this docking grid was selected around the predicted active site. From the ten ligands, one best docked result for each target was selected and provided in Table 2 with their ligand structure, binding energy, ligand efficiency and number of hydrogen bonds. The binding orientation of all five complexes is shown in Figure 3.

**Figure 4:**
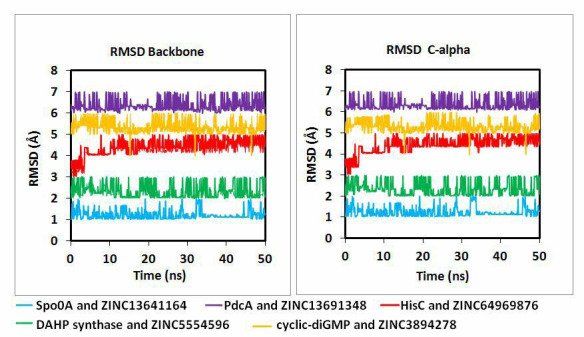
RMSD plots for Molecular dynamics studies for all five protein ligand complexes.

The best compounds that bind effectively with the target were further analysed for their stability and interaction using molecular dynamics (MD). To reduce error and artifacts, the experiment was carried out in triplicates for 50 ns that aid in obtaining substantial and reproducible MD results. All the protein ligand complexes were placed in cubic box neutralizing system. The system contains TIP3P water and is simulated for 50 ns at constant 300K temperature and 1 bar pressure. To evaluate the stability of the system, RMSD (root mean square deviation) and RMSF (root mean square fluctuation) were calculated using gmx_rms and gmx_rmsf commands. In Figure 4, RMSD graphs for backbone and alpha carbon shows that all trajectories reach equilibrium. It is one of the key parameters to investigate the insight into protein backbone and ligand stability during the MD simulation. The consistency of the protein-ligand complexes in dynamic states is explained by the consistent deviation or low variation of the RMSD value in Figure 4.

To explore the insight protein backbone and ligand stability during the MD simulation, the protein backbone RMSD is one of the important parameters. As we run the dynamics in triplicates, the average RMSD for backbone and C alpha is almost the same. It was seen that the HisC backbone bound with ZINC64969876 deviated slightly in the initial stage of simulation, and afterwards it achieved consistency around an RMSD of 4 nm till the end of the simulation. Although the lowest RMSD is found to be of Spo0A and ZINC13641164 which is around 1.5nm and it declines to 1nm around 35 to 45 ns. It might be due to more conformational changes of ZINC13641164 inside the Spo0A binding pocket. Hence, from the above data and observations it was clear that all the protein ligand complexes achieved stability in the dynamic states.

**Figure 5:**
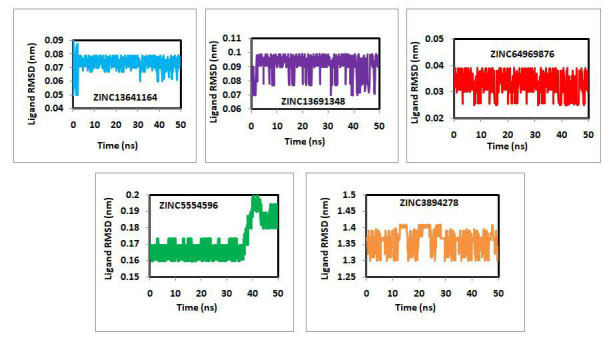
Ligand RMSD of ZINC13641164, ZINC13691348, ZINC64969876, ZINC5554596 and ZINC3894278.

The ligand RMSD data against the time of simulation of ZINC13641164, ZINC13691348, ZINC64969876, ZINC5554596 and ZINC3894278 was plotted and is given in Figure 5. Almost all ligands remained steady in dynamic states throughout the simulation, with a few exceptions. Ligand ZINC5554596 was seen to be steady till 40ns and suddenly the RMSD was increased from around 0.17 to 2.0 nm and further attained steadiness till the end of the simulation. This may be due to change in the molecule's conformational orientation. The differences between the maximum and average RMSD can provide insight into the overall molecular deviation from the mean position. The values are found to be 0.17, 0.09, 0.07, 0.028 and 0.5 for ZINC13641164, ZINC13691348, ZINC64969876, ZINC5554596 and ZINC3894278, respectively. Based on the above low values and consistent variation of ligand RMSD suggested, the ligands are stable inside the active sites of the target. During MD run, all ligand remain bound to their respective targets throughout the simulation period.

**Figure 6:**
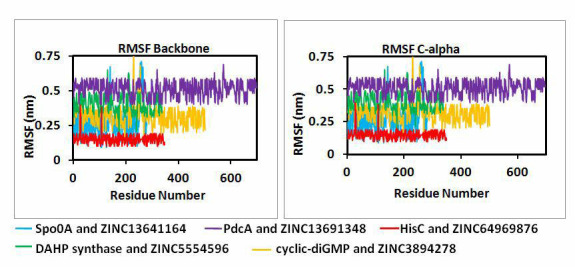
RMSF plots for Molecular dynamics studies done for all five protein ligand complexes.

We have also plotted RMSF graph of individual amino acid residues to check the fluctuations in the amino acid residues of the active sites. In Figure 6 the RMSF for backbone and C- alpha shows that amino acids at active sites are not much fluctuating. However, a little fluctuation is seen at active site of Spo0A and cyclic-diGMP, this may be because more number of residues is participating in bringing about more fluctuation to the system. For Spo0A residues Thr255 and Lys256 and for cyclic-diGMP residues Asn229 and Phe248 are showing fluctuations. Except this fluctuation, the RMSF graphs suggest that the interactions of protein-ligand complexes were maintained during the MD run.

**Figure 7:**
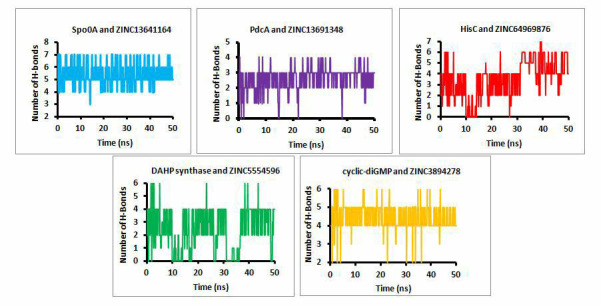
Number of hydrogen bonds formed between protein and their respective ligands during simulation.

The plots for number of hydrogen bonds formed during simulation are also plotted. Figure 7 shows the number of hydrogen bonds formed between each protein ligand complex. Average number of hydrogen bonds formed during simulation for Spo0A and ZINC13641164 is 6, for PdcA and ZINC13691348 is 3, for HisC and ZINC64969876 is 5, for DAHP synthase and ZINC5554596 is 4 and for cyclic-diGMP and ZINC3894278 is 5.

**Figure 8:**
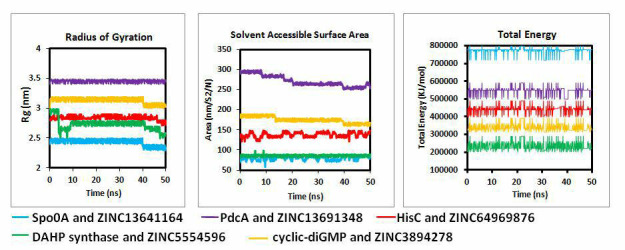
Radius of Gyration, Solvent accessible surface area (SASA) and total energy plots for all the 5 complexes over 50 ns of simulation time.

In addition, plots for radius of gyration (Rg), solvent accessible surface area (SASA) and total energy are also plotted for all five protein ligand complexes (Figure 8). Radius of gyration is defined as the root mean square distance between each atom in a structure and its centre of mass. As seen from the plot, with the exception of PdcA, Rg is almost always decreasing, suggesting that ligand binding aids in stabilising and achieving compactness of the protein molecule. SASA is also considered as the important factor for determining protein folding and stability. It is measured as the surface area of protein that is accessible to solvent. SASA suggests the impact of ligand binding on the profile of amino acids at the protein surface. From the plot, it is clear that PdcA, HisC, cyclic-di GMP shows decreasing SASA which signifies that they undergo important structural changes upon binding of the ligand. The total energy plot for all the protein ligand complexes is same across the simulation suggesting the stability of the complexes.

After *in silico* studies, *in vitro* compounds are tested on the four isolates using agar well diffusion assay and Minimum Inhibitory Concentration (MIC). The compounds were ordered from (www.mcule.com). All the five compounds show zone of inhibition with each of the four isolates, as shown in Table 3. The negative control taken as DW does not show any zone of inhibition. As seen from the table, the zone of inhibition increases with the increase in concentration of the compound. All compounds show comparable inhibition except ZINC64969876 which is showing less inhibition.

**Figure 9:**
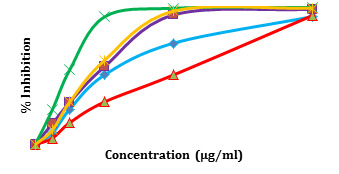
Percentage inhibition of *C. difficile* at different concentrations of compounds.

The minimum inhibitory concentrations of all the five compounds on each of the four isolates are measured. Their percentage inhibition is calculated and is shown in Figure 9. From the graph, it can be noted that compound ZINC13641164, ZINC13691348 and ZINC3894278 shows 50% inhibition at 25 µg/ml, and compound ZINC5554596 shows 50% inhibition at 12.5µg/ml. The compound ZINC64969876 shows 50% inhibition at highest concentration, 50µg/ml. Through *in silico* studies, we have concluded that five potent compounds namely, ZINC13641164, ZINC13691348, ZINC64969876, ZINC5554596 and ZINC3894278 are capable of inhibiting *C. difficile*. On further *in vitro* studies, the compounds ZINC13641164, ZINC13691348, ZINC3894278 and ZINC5554596 are found to be most suitable. All these compounds have been reported for the first time as antimicrobial agents and need further in vivo studies before being used as anti-clostridial drugs. Table 4 shows the list of compounds with their smiles that were identified as inhibitors of *C. difficile*.

## Conclusion:

The rapid emergence of *C. difficile* virulent strains and development of antibiotic resistance in *C.difficile* creates a challenge to rapidly identify more drugs to treat this pathogen. In addition, high recurrence rate also makes this pathogen an attention seeker pathogen. As an urgent need, we have used structural biology approach to find the new lead molecules against *C difficile*. It is the cheapest, quickest and most reliable method to discover drugs against pathogen and has been used earlier for other pathogens. In our computer-aided drug design method, we have focused on the five key proteins that have been identified as *C. difficile* therapeutic targets. For all the targets, we conclude the best lead compounds that can impair the functions of these proteins and affect the survival of the pathogen. The lead compounds are ZINC1364116 for Spo0A, ZINC13691348 for PdcA, ZINC64969876 for HisC, ZINC5554596 for DAHP synthase and ZINC3894278 for cyclic-diGMP. However, clinical trials are required to use these lead compounds as anti-clostridial agents.

## Funding:

There are no relevant financial or non-financial competing interests to report. This research received no specific grant from any funding agency in the public, commercial or not-for-profit sectors.

## Figures and Tables

**Table 1:** Different filters and their threshold used to find the drug likeliness property of the compounds.

**Name of Filters**	**Parameters**
Lipinski Rule	Molecular Weight <=500D, LogP<=5, H-Bond Donor<=5, H-Bond acceptor<=10
Ghose Filter	Molecular Weight = 160 to 480D, LogP=-0.4 to 5.6, Atom Count= 20 to70, Refractivity= 40 to 130
CMC-50 Like Rule	Molecular Weight = 230 to 390D, LogP= 1.3 to 4.1, Atom Count=30 to 55, Refractivity= 70 to 110
Veber Rule	Rotatable Bond<=10, Polar surface area<=140
MDDR Like Rule	No of Rings >= 3, No. of Rigid bonds>=18, Rotatable Bond>=6
BBB Likeness Rule	Molecular Weight <=400D, H Bonds(total)<=8, No. acids=0
QED Filter	Weighted QED>=0.5, Unweighted QED>=0.5

**Table 2:** Site -specific Molecular docking score of the five docked complexes

**Target**	**Binding Energy (kcal/mol)**	**Ligand efficiency (kcal/mol)**	**Number of H Bonds**
Sporulation transcription factor (Spo0A)	-9.59	-0.12	5 (Lys193, Lys256, Ala266, Thr255, Pro141)
Cyclic-di-GMP phosphodiesterase (PdcA)	-10.36	-0.003	3 (Asn537, Thr571, Glu569)
Histidinol-phosphate transaminase (HisC)	-9.42	0.01	4 (Asn27, Phe108, Glu28, Gly29)
3-deoxy-7-phosphoheptulonate synthase (DAHP synthase)	-8.96	-0.08	4 (Arg186, Gly209, Ala212, Leu210)
Bifunctional diguanylate cyclase/ phosphodiesterase (cyclic-diGMP)	-8.17	-0.2	4 (Glu255, Phe248,Asn229, Lys251)

**Table 3:** Zone of inhibition (in mm) measured for all the four isolates at different concentrations of compounds.

**Compound**	**Concentration**	**Isolate 1**	**Isolate 2**	**Isolate 3**	**Isolate 4**
ZINC13641164	50 µg/ml	5.1±0.1	7.2±0.2	7.8±0.1	7.2±0.5
	100 µg/ml	7.0±0.1	9.2±0.4	9.5±0.3	9.3±0.2
	150 µg/ml	10.8±0.3	10.3±0.1	10.4±0.2	10.8±0.1
	200 µg/ml	13.3±0.3	13.6±0.2	13.8±0.1	13.5±0.4
	250 µg/ml	15.5±0.4	15.4±0.2	16.5±0.3	16.2±0.2
ZINC13691348	50 µg/ml	4.5±0.5	5.2±0.2	4.6±0.1	5.5±0.2
	100 µg/ml	6.1±0.2	6.3±0.2	6.2±0.2	6.2±0.3
	150 µg/ml	8.4±0.2	8.0±0.1	8.0±0.4	8.4±0.2
	200 µg/ml	12.5±0.1	12.2±0.2	12.2±0.1	12.5±0.3
	250 µg/ml	15.3±0.2	15.3±0.1	14.8±0.4	14.6±0.2
ZINC64969876	50 µg/ml	3.2±0.2	4.2±0.2	4.3±0.2	3.9±0.1
	100 µg/ml	6.5±0.1	6.1±0.3	5.8±0.1	5.4±0.1
	150 µg/ml	7.3±0.2	7.5±0.2	7.4±0.3	7.1±0.2
	200 µg/ml	8.8±0.2	8.3±0.1	8.8±0.4	8.2±0.1
	250 µg/ml	10.1±0.2	9.8±0.5	10.8±0.3	10.5±0.2
ZINC5554596	50 µg/ml	5.2±0.2	5.0±0.3	6.6±0.2	4.8±0.1
	100 µg/ml	7.2±0.3	7.5±0.2	7.2±0.3	7.5±0.2
	150 µg/ml	10.5±0.2	10.7±0.1	10.1±0.5	10.7±0.3
	200 µg/ml	13.5±0.5	11.6±0.3	11.9±0.2	7.8±0.2
	250 µg/ml	15.2±0.4	13.2±0.2	14.2±0.5	15.0±0.1
ZINC3894278	50 µg/ml	4.5±0.5	4.2±0.3	5.2±0.3	5.6±0.3
	100 µg/ml	6.1±0.2	6.4±0.1	6.2±0.1	7.5±0.5
	150 µg/ml	8.3±0.2	9.2±0.0	7.8±0.6	8.9±0.2
	200 µg/ml	9.5±0.1	10.8±0.1	9.2±0.4	9.7±0.2
	250 µg/ml	11.3±0.1	13.5±0.2	11.5±0.5	12.5±0.3

**Table 4:** List of compounds that were identified as inhibitors of *C.difficile*

**ZINC ID**	**SMILES**	**Mol wt**	**Log P**	**H-bond donors**	**H-bond acceptors**	**Apolar desolvation**	**Polar desolvation**
ZINC13641164	Cc1onc(C(=O)NCCCn2cccn2) c1COc1ccc([N+](=O)[O-])cc1	385.38	2.487	1	8	7.39	-16.41
ZINC13691348	CN1CCN(C2=NC(=O)[C@@H] (C#N)C3(CCN(Cc4ccccc4)CC3) N2)CC1	380.496	0.894	3	5	10.66	-92.24
ZINC64969876	Cc1cccc(Cc2nc(CCNS(=O)(=O) c3c(C)noc3C)n[nH]2)c1	375.454	1.83	2	6	5.79	-19.05
ZINC5554596	CCCCCOc1cc(O)c2c(=O)cc(-c3ccccc3)oc2c1	324.376	4.735	1	4	8.78	-22.19
ZINC3894278	O=c1ccc2cc(O[C@@H]3O [C@@H](CO)[C@@H](O) [C@@H](O)[C@@H]3O)c (O)cc2o1	340.284	-1.323	5	9	-5.11	-15.51

## References

[1] Pal R, Seleem MN (2022). Plos one.

[2] D'Silva KM (2021). Clin Microbiol Infect..

[3] Marra AR (2020). JAMA Netwopen..

[4] Naclerio GA (2020). J Med Chem..

[5] Abou Chakra CN (2014). PloS one..

[6] Tomkovich S (2019). mSphere..

[7] Cortés P (2021). Rom. J.Intern. Med..

[8] Lin CY (2022). Microbiol Spectr..

[9] Hussack G (2023). Front Microbiol..

[10] Burns DA, Minton NP (2011). J.Microbial. Methods.

[11] Spigaglia P (2022). Anaerobe..

[12] Sandhu A (2020). Emerg Infect. Dis..

[13] Sholeh M (2020). Antimicrob. Resist. Infect. Control..

[14] Phanchana M (2020). Sci Rep..

[15] Louie TJ (2011). New England J. Med..

[16] Johnson S (2014). Clin. Infect. Dis..

[17] Abutaleb NS, Seleem M. (2020). Antimicrob. Agents chemother..

[18] Sholeh M (2020). Antimicrob. Resist. Inf. Contr..

[19] Mohammad AW (2023). Cureus..

[20] Okura Y (2022). DEN Open..

[21] Chung YS (2022). Onco Targets Ther..

[22] Golchha NC (2022). Drug Target Insights.

[23] Webb B, Sali A. (2016). Curr. Prot.Bioinfor..

[24] Reddy AR (2015). Int. J. Pharm. Sci. Rev. Res.

[25] Colovos C, Yeates TO (1993). Prot. Sci..

[26] Lüthy R (1992). Nature.

[27] Laskowski RA (1993). J. Appl. Crystallogr..

[28] Sterling T, Irwin JJ (2015). J. Chem. inform.Mod..

[29] https://niper.gov.in/pi_dev_tools/DruLiToWeb/DruLiTo_index.html.

[30] Banerjee P (2018). Nucl acids res..

[31] Wolf LK. (2009). Chem Eng News..

[32] Dallakyan S, Olson AJ (2015). Chem. biol meth prot..

[33] Forli S (2016). Nat proto..

[34] Chatterjee A (2011). Int. J. Curr. Eng. Technol..

[35] Yuan S (2017). Wiley Inter discipl. Rev.: Comput. Mol. Sci..

[36] Van Der Spoel D (2005). J. Comput. Chem..

[37] Vanommeslaeghe K (2010). J. Comput. Chem..

[38] Zoete V (2011). J. Comput. Chem..

[39] https://pubmed.ncbi.nlm.nih.gov/17517836/.

[40] Giménez BG (2010). Int. J. Pharm. Sci..

[41] Khanna V, Ranganathan S (2011). J. Cheminform..

